# Isolation within Isolation Hospitals

**Published:** 1900-05-05

**Authors:** 


### Hospital Clinics and Medical Progress.

### ISOLATION WITHIN ISOLATION HOSPITALS.

One of the most anxious problems which confront the managers of fever hospitals is how to prevent the patients brought in with one kind of infectious disease being attacked by another. That this unfortunate occurrence does take place in a not inconsiderable number of cases is undoubtedly the fact, and it is by no means easy to see how it is to be prevented. It is one of the "difficulties in the administration of fever hospitals," to which Dr. Goodall referred in a paper with that title which he recently read before the Society of Medical Officers of Health. What seems to us one of the most important and practical points to which he drew attention in this paper is that it is impossible to prevent the introduction of various kinds of infection into a fever hospital, and that it is a better plan to be prepared at each hospital to deal with whatever cases may turn up than to attempt to keep separate hospitals exclusively for each disease. The frequent occurrence of post-scarletinal diphtheria, and the occasional occurrence of post-diphtheritic scarlet fever, have led some people to maintain that one should treat these diseases in different hospitals just as one separates small-pox from other kinds of fever. And if one could be sure that before being sent to hospital the exact nature of each case could be made certain, and that no case should ever bring in two infections, there would be much to be said in favour of such a plan. Unfortunately, we cannot be sure of this. The very essence of "isolation," as a means of preventing the spread of epidemics, lies in early removal of infected cases; and early removal almost of necessity involves the removal of a certain number of cases in which the exact nature of the fever still remains in doubt. Especially does this difficulty arise as between "sore throat," diphtheria, and scarlet fever. But all sorts of pyrexial diseases are apt to be sent to fever hospitals, and they cannot be refused off hand. Dr. Goodall says that measles, whooping cough, and mumps, the infection of which had doubtless been contracted by patients before their admission, have all broken out from time to time. Moreover, it is not rare for a case admitted with diphtheria to develop scarlet fever under circumstances excluding the supposition of infection in the hospital, while cases of diphtheria have also communicated scarlet fever to others in the ward, the diseases having coexisted, but the angina having been concealed by the exudation, and the rash having been absent. In the great majority of these mixed cases the patients were admitted as cases of diphtheria, and in a few as cases of scarlet fever. However much one might build separate hospitals for each different disease such cases would carry both infections into whichever hospital they entered. There is no doubt that Dr. Goodall is right in saying that to guard against such contingencies every fever hospital ought to be constructed with numerous small wards, not only for isolation and observation but for the separation of different diseases, and he seems to have reason on his side in saying that there is no advantage in providing separate hospitals for different fevers seeing that other diseases will always creep in. This does not apply only to fever hospitals. All hospitals should be provided with a considerable proportion of small wards so arranged that by their use a fairly complete degree of isolation can be obtained for any cases which it is desired to cut off from the rest. Such wards are expensive to build and expensive to maintain, and they sadly break into the symmetry of plan which so many architects love; but they are essential if the patients are to be protected from all avoidable risks.

